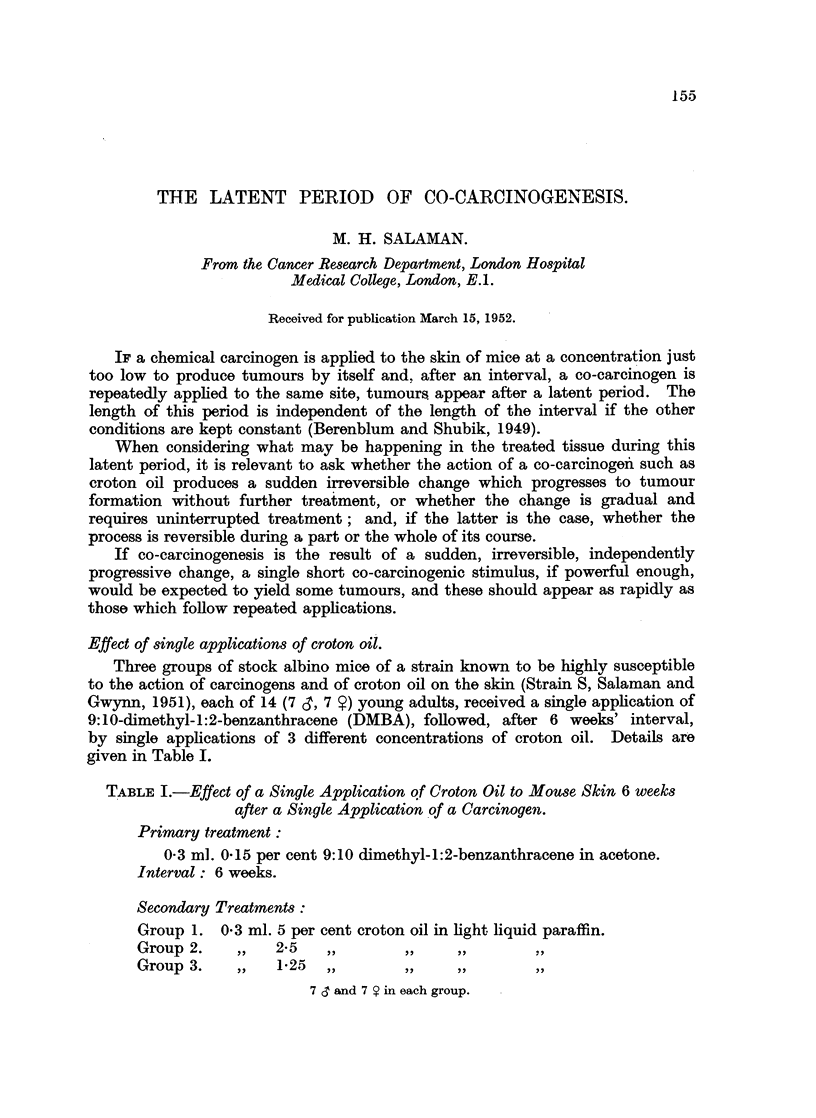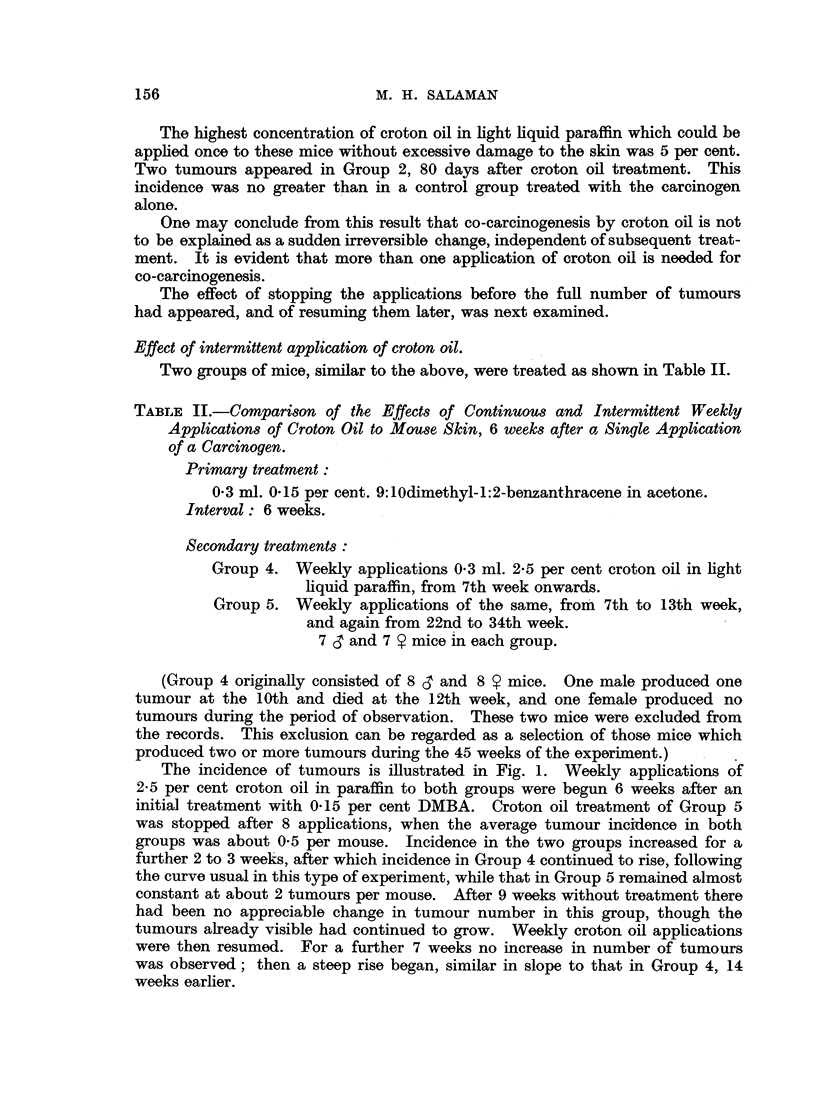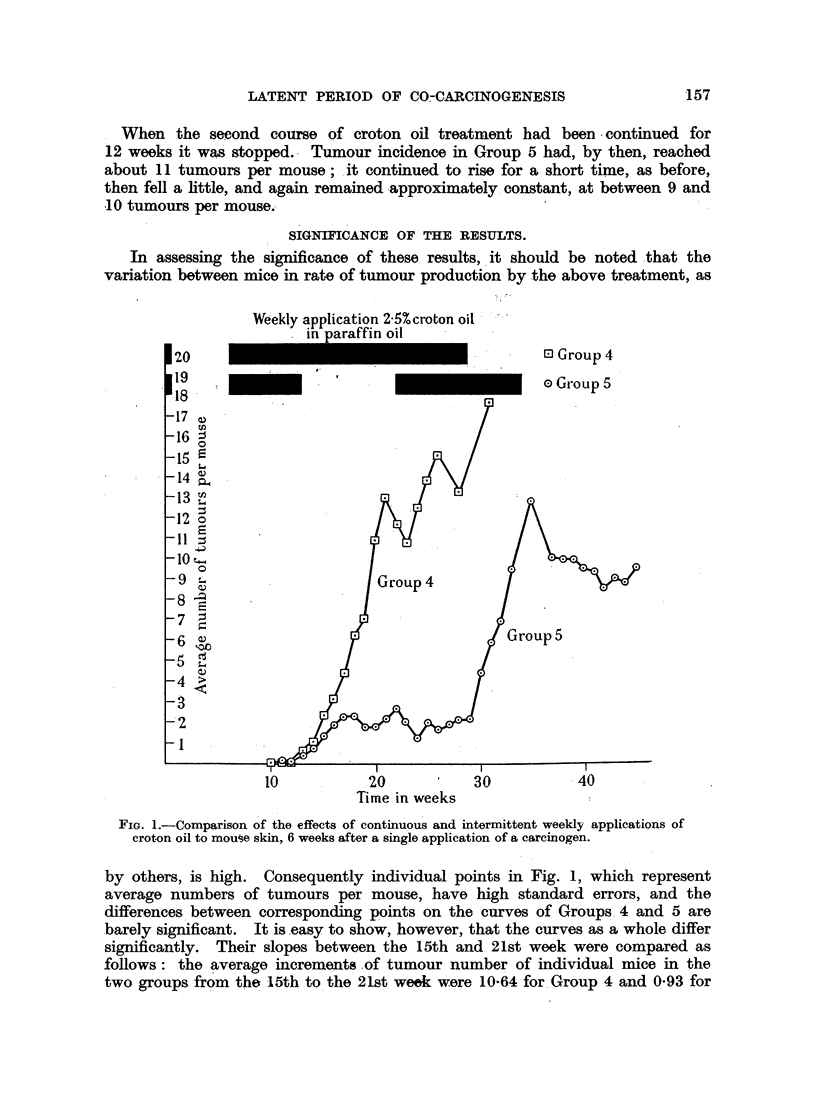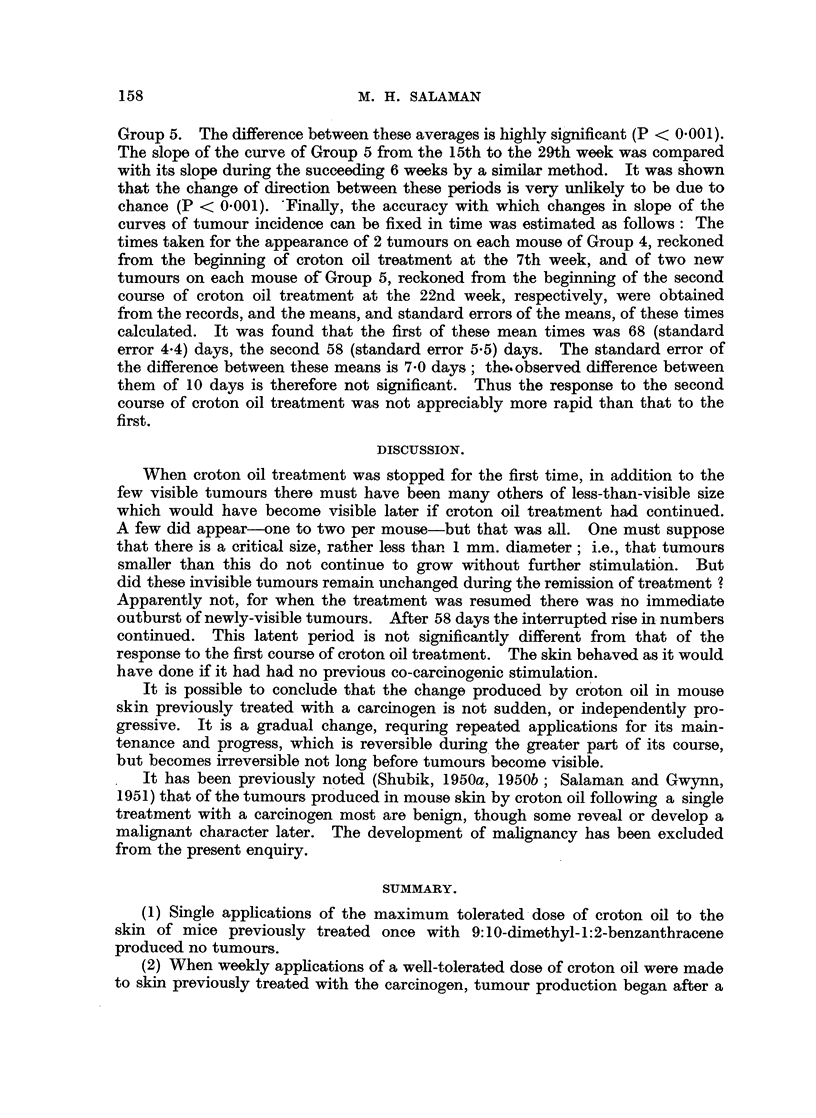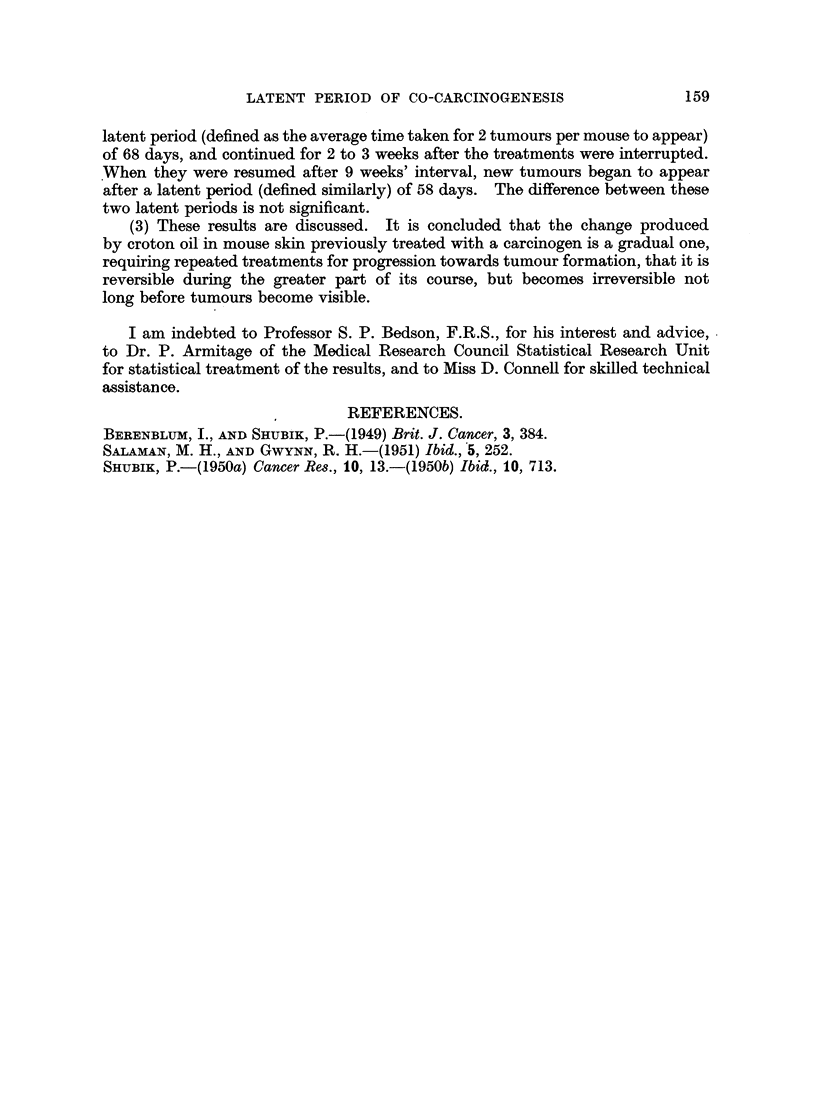# The Latent Period of Co-Carcinogenesis

**DOI:** 10.1038/bjc.1952.18

**Published:** 1952-06

**Authors:** M. H. Salaman


### THE LATENT PERIOD OF CO-CARCINOGENESIS.

M. H. SALAMAN.

*From the Cancer Research Department, London Hospital Medical College, London, E.1.*

Received for publication March 15, 1952.

IF a chemical carcinogen is applied to the skin of mice at a concentration just too low to produce tumours by itself and, after an interval, a co-carcinogen is repeatedly applied to the same site, tumours appear after a latent period. The length of this period is independent of the length of the interval if the other conditions are kept constant (Berenblum and Shubik, 1949).

When considering what may be happening in the treated tissue during this latent period, it is relevant to ask whether the action of a co-carcinogen such as croton oil produces a sudden irreversible change which progresses to tumour formation without further treatment, or whether the change is gradual and requires uninterrupted treatment; and, if the latter is the case, whether the process is reversible during a part or the whole of its course.

If co-carcinogenesis is the result of a sudden, irreversible, independently progressive change, a single short co-carcinogenic stimulus, if powerful enough, would be expected to yield some tumours, and these should appear as rapidly as those which follow repeated applications.

*Effect of single applications of croton oil.*

Three groups of stock albino mice of a strain known to be highly susceptible to the action of carcinogens and of croton oil on the skin (Strain S, Salaman and Gwynn, 1951), each of 14 (7 ♂, 7 ♀) young adults, received a single application of 9:10-dimethyl-1:2-benzanthracene (DMBA), followed, after 6 weeks' interval, by single applications of 3 different concentrations of croton oil. Details are given in Table I.

TABLE I.—*Effect of a Single Application of Croton Oil to Mouse Skin 6 weeks after a Single Application of a Carcinogen.*

*Primary treatment :*

0·3 ml. 0·15 per cent 9:10 dimethyl-1:2-benzanthracene in acetone.

*Interval :* 6 weeks.

*Secondary Treatments :*

| | | | | | | |
|---|---|---|---|---|---|---|
| Group 1. | 0·3 ml. | 5 per cent | croton oil | in light | liquid | paraffin. |
| Group 2. | ,, | 2·5 | ,, | ,, | ,, | ,, |
| Group 3. | ,, | 1·25 | ,, | ,, | ,, | ,, |

7 ♂ and 7 ♀ in each group.

The highest concentration of croton oil in light liquid paraffin which could be applied once to these mice without excessive damage to the skin was 5 per cent. Two tumours appeared in Group 2, 80 days after croton oil treatment. This incidence was no greater than in a control group treated with the carcinogen alone.

One may conclude from this result that co-carcinogenesis by croton oil is not to be explained as a sudden irreversible change, independent of subsequent treatment. It is evident that more than one application of croton oil is needed for co-carcinogenesis.

The effect of stopping the applications before the full number of tumours had appeared, and of resuming them later, was next examined.

*Effect of intermittent application of croton oil.*

Two groups of mice, similar to the above, were treated as shown in Table II.

TABLE II.—*Comparison of the Effects of Continuous and Intermittent Weekly Applications of Croton Oil to Mouse Skin,* 6 *weeks after a Single Application of a Carcinogen.*

*Primary treatment :*

0·3 ml. 0·15 per cent. 9:10dimethyl-1:2-benzanthracene in acetone.

*Interval :* 6 weeks.

*Secondary treatments :*

Group 4. Weekly applications 0·3 ml. 2·5 per cent croton oil in light liquid paraffin, from 7th week onwards.

Group 5. Weekly applications of the same, from 7th to 13th week, and again from 22nd to 34th week.

7 ♂ and 7 ♀ mice in each group.

(Group 4 originally consisted of 8 ♂ and 8 ♀ mice. One male produced one tumour at the 10th and died at the 12th week, and one female produced no tumours during the period of observation. These two mice were excluded from the records. This exclusion can be regarded as a selection of those mice which produced two or more tumours during the 45 weeks of the experiment.)

The incidence of tumours is illustrated in Fig. 1. Weekly applications of 2·5 per cent croton oil in paraffin to both groups were begun 6 weeks after an initial treatment with 0·15 per cent DMBA. Croton oil treatment of Group 5 was stopped after 8 applications, when the average tumour incidence in both groups was about 0·5 per mouse. Incidence in the two groups increased for a further 2 to 3 weeks, after which incidence in Group 4 continued to rise, following the curve usual in this type of experiment, while that in Group 5 remained almost constant at about 2 tumours per mouse. After 9 weeks without treatment there had been no appreciable change in tumour number in this group, though the tumours already visible had continued to grow. Weekly croton oil applications were then resumed. For a further 7 weeks no increase in number of tumours was observed ; then a steep rise began, similar in slope to that in Group 4, 14 weeks earlier.

When the second course of croton oil treatment had been continued for 12 weeks it was stopped. Tumour incidence in Group 5 had, by then, reached about 11 tumours per mouse ; it continued to rise for a short time, as before, then fell a little, and again remained approximately constant, at between 9 and 10 tumours per mouse.

SIGNIFICANCE OF THE RESULTS.

In assessing the significance of these results, it should be noted that the variation between mice in rate of tumour production by the above treatment, as

FIG. 1.—Comparison of the effects of continuous and intermittent weekly applications of croton oil to mouse skin, 6 weeks after a single application of a carcinogen.

by others, is high. Consequently individual points in Fig. 1, which represent average numbers of tumours per mouse, have high standard errors, and the differences between corresponding points on the curves of Groups 4 and 5 are barely significant. It is easy to show, however, that the curves as a whole differ significantly. Their slopes between the 15th and 21st week were compared as follows : the average increments of tumour number of individual mice in the two groups from the 15th to the 21st week were 10·64 for Group 4 and 0·93 for

Group 5. The difference between these averages is highly significant ($P < 0{\cdot}001$). The slope of the curve of Group 5 from the 15th to the 29th week was compared with its slope during the succeeding 6 weeks by a similar method. It was shown that the change of direction between these periods is very unlikely to be due to chance ($P < 0{\cdot}001$). Finally, the accuracy with which changes in slope of the curves of tumour incidence can be fixed in time was estimated as follows: The times taken for the appearance of 2 tumours on each mouse of Group 4, reckoned from the beginning of croton oil treatment at the 7th week, and of two new tumours on each mouse of Group 5, reckoned from the beginning of the second course of croton oil treatment at the 22nd week, respectively, were obtained from the records, and the means, and standard errors of the means, of these times calculated. It was found that the first of these mean times was 68 (standard error 4·4) days, the second 58 (standard error 5·5) days. The standard error of the difference between these means is 7·0 days; the observed difference between them of 10 days is therefore not significant. Thus the response to the second course of croton oil treatment was not appreciably more rapid than that to the first.

### DISCUSSION.

When croton oil treatment was stopped for the first time, in addition to the few visible tumours there must have been many others of less-than-visible size which would have become visible later if croton oil treatment had continued. A few did appear—one to two per mouse—but that was all. One must suppose that there is a critical size, rather less than 1 mm. diameter; i.e., that tumours smaller than this do not continue to grow without further stimulation. But did these invisible tumours remain unchanged during the remission of treatment? Apparently not, for when the treatment was resumed there was no immediate outburst of newly-visible tumours. After 58 days the interrupted rise in numbers continued. This latent period is not significantly different from that of the response to the first course of croton oil treatment. The skin behaved as it would have done if it had had no previous co-carcinogenic stimulation.

It is possible to conclude that the change produced by croton oil in mouse skin previously treated with a carcinogen is not sudden, or independently progressive. It is a gradual change, requring repeated applications for its maintenance and progress, which is reversible during the greater part of its course, but becomes irreversible not long before tumours become visible.

It has been previously noted (Shubik, 1950*a*, 1950*b*; Salaman and Gwynn, 1951) that of the tumours produced in mouse skin by croton oil following a single treatment with a carcinogen most are benign, though some reveal or develop a malignant character later. The development of malignancy has been excluded from the present enquiry.

### SUMMARY.

(1) Single applications of the maximum tolerated dose of croton oil to the skin of mice previously treated once with 9:10-dimethyl-1:2-benzanthracene produced no tumours.

(2) When weekly applications of a well-tolerated dose of croton oil were made to skin previously treated with the carcinogen, tumour production began after a latent period (defined as the average time taken for 2 tumours per mouse to appear) of 68 days, and continued for 2 to 3 weeks after the treatments were interrupted. When they were resumed after 9 weeks' interval, new tumours began to appear after a latent period (defined similarly) of 58 days. The difference between these two latent periods is not significant.

(3) These results are discussed. It is concluded that the change produced by croton oil in mouse skin previously treated with a carcinogen is a gradual one, requiring repeated treatments for progression towards tumour formation, that it is reversible during the greater part of its course, but becomes irreversible not long before tumours become visible.

I am indebted to Professor S. P. Bedson, F.R.S., for his interest and advice, to Dr. P. Armitage of the Medical Research Council Statistical Research Unit for statistical treatment of the results, and to Miss D. Connell for skilled technical assistance.

### REFERENCES.

Berenblum, I., and Shubik, P.—(1949) *Brit. J. Cancer*, **3**, 384.
Salaman, M. H., and Gwynn, R. H.—(1951) *Ibid.*, **5**, 252.
Shubik, P.—(1950*a*) *Cancer Res.*, **10**, 13.—(1950*b*) *Ibid.*, **10**, 713.